# Influence of the Geometrical Features of the Cutting Edges of Abrasive Grains on the Removal Efficiency of the Ti6Al4V Titanium Alloy

**DOI:** 10.3390/ma15186189

**Published:** 2022-09-06

**Authors:** Łukasz Rypina, Dariusz Lipiński, Kamil Banaszek, Wojciech Kacalak, Filip Szafraniec

**Affiliations:** 1 Faculty of Mechanical Engineering, Koszalin University of Technology, Racławicka 15, 75-620 Koszalin, Poland; 2 Doctoral School, Koszalin University of Technology, Racławicka 15, 75-620 Koszalin, Poland

**Keywords:** grinding, abrasive grain, material removal, efficiency, rake angle, opening angle, apex angle, cutting edge, cutting blade, titanium alloy, finite elements methods (FEM)

## Abstract

The shape of the cutting blades of the abrasive grains has an influence on the material separation process in the machining zone. The paper analyzes the influence of the geometrical parameters of the abrasive grains (rake angle *γ*, apex angle *ε*, opening angle *α*), as well as width *b_z_* and length *b_b_* of the cutting zone on the material removal efficiency. The material removal efficiency was determined taking into account the volume of the removed material *V_G_* and the volume of lateral piles-up *V_R_*. The analyses were carried out on the basis of the results of experimental and simulations using the finite element method. The relationship between the selected geometric parameters characterizing the cutting zone and the coefficient characterizing the efficiency of the material removal process was determined. A strong influence of the opening angle α as well as the width *b_z_* and length *b_b_* of the cutting zone on the material removal process by abrasive grain was demonstrated. It was observed that the wide cutting edge, and thus the large opening angle *α* of the grain, reduced the size of the pile-ups and more effectively removed the chip material.

## 1. Introduction

Increasing requirements for mechanical parts concern their durability, reliability and good surface quality. Hence, the final machining should be performed with appropriately selected parameters and conditions [1,2]. Titanium alloys are difficult to cut. During the machining of titanium alloys, low thermal conductivity is the reason that approximately 15% of the heat can be accumulated in the cutting edge [3]. This requires improved machining potential of the grinding tools, which should also have high productivity and wear resistance, and should allow for the reduction in thermal damages on the ground surfaces [4,5,6].

To explain the phenomena of material removal processes, the research should be carried out for three different stages: (i) rubbing, (ii) ploughing, (iii) chip formation. During the rubbing stage, the elastic deformation occurs the most frequently. During ploughing, the grain moves deeper into the material and mainly plastic deformation occurs on the rake face of the grain. Furthermore, the material moves to the sides and forms the pile-up. During the third stage, the chip formation occurs as a result of continuous increase in the depth of the cut [7,8].

Early works were based on the assumptions that the single-grain scratch test should be treated similarly to the orthogonal cutting model, developed by Merchant [9,10]. In orthogonal turning, chip formation occurs; simultaneously, the material moves along the shear plane. The separated chip moves almost entirely parallel to the surface of the blade, which has a positive rake angle. Almost all of the energy is used for the chip formation process [11]. In order to move closer to the machining conditions during the cutting with a single abrasive grain, the tests were carried out for blades with negative rake angles [12]. It was proven that the increase in the negative rake angle of the cutting edge increased the cutting forces and the specific energy. Modeling of the cutting processes with a single abrasive grain is difficult, because of the large number and complexity of the phenomena occurring in the cutting zone. The shape of the contact zone changes as the cutting depth increases. This also affects the parameters of the material flow [13,14,15].

Doman et al. [16,17] presented one of the first results of the cutting processes modeling with single cutting edge, using the finite elements method (FEM). Mathematical models, methods for defining boundary conditions as well as methods for choosing the constitutive material models, were discussed. Machining with single abrasive grain was carried out to verify simulation results [16,17]. The rubbing and plowing phases, which are typical for the process, were analyzed. Special attention was given to the experimental validation of the model. Good agreement was achieved between the normal and tangential force comparisons. The surface features of the scratches (width and shape) were consistent with the observed experimental results.

Due to the complex nature of the abrasive machining, the experimental studies are insufficient for a proper understanding of the material removal phenomena. Many researchers [13,18,19,20,21,22] used FEM to explain the influence of the abrasive grains’ geometrical parameters on the plastic material flows. In order to study abrasive grain wear and understand the material removal processes, Zhao et al. [18,19] conducted single-grain grinding of Ti-6Al-4V alloys. The proposed approach was to track grains and scratches by means of 3D reconstruction. The grinding force ratio, material removal efficiency and grain wear characteristics were discussed.

In the work by Dai et al. [20], the influence of the cutting edges geometry of diamond grains on the cutting process efficiency was investigated. The variability of the scratching process, grinding force, the level of material accumulation as well as specific grinding energy, were investigated in various conditions. The results have shown that the straight (chisel-shaped) cutting edge allows for reducing the side material flows and allows for increasing material removal efficiency. Otherwise, the inclined cutting edges increase the side material flows. The grain geometry determines the cutting process. For example, the increase in the negative rake angle leads to greater plastic deformation [21] and has an influence on chip formation [13,22,23]. Gao et al. [24] presented the influence of the active abrasive grains orientation on the performance and material removal rates during the honing process. By means of FEM simulation, it was possible to analyze the variation of material removal, material removal rate, surface roughness and honing force.

In this article, the geometrical parameters of the cutting edges and their influence on the separation of workpiece material in the form of chips and lateral pile-ups, are discussed. Experiments on the cutting process with a single abrasive grain and numerical FEM simulations were carried out. On the basis of the test results, a material removal rate was determined for abrasive grains with different geometrical parameters of the cutting edges. During the analyses, the volume of the groove and the volume of the lateral pile-ups were taken into account. Additionally, the influence of the geometrical parameters of the abrasive grain cutting edges (opening angle *α*, apex angle *ε* and rake angle *γ*), as well as the width *b_z_* and length *b_b_* of the cutting zone on the value of the cutting efficiency factor *k_r_*, was determined.

## 2. Materials and Methods

During grinding with a single grain, the material displacement analysis is difficult to perform using experimental measurement techniques. Real-time registration of the processes occurring near the micro-blades of abrasive grains is almost impossible. We present here a numerical model which allows to research the influence of the abrasive grains’ geometrical parameters on the material removal processes. The results are validated experimentally.

### 2.1. Experiment and Model Validation

**Figure 1:**
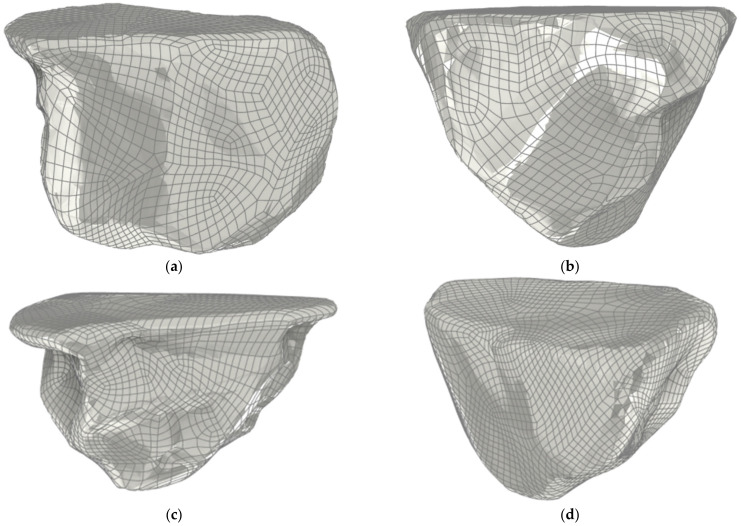
Alumina grains 97A with size F20, applied for numerical model validation (**a**) and numerical simulations (**b**–**d**).

In the presented study, the alumina grains 97A with size F20 were used. The grain presented in Figure 1a was used for simulation and experiment. The rest of the abrasive grain models (Figure 1b–d) were used only for numerical simulations of the material removal process.

**Figure 2:**
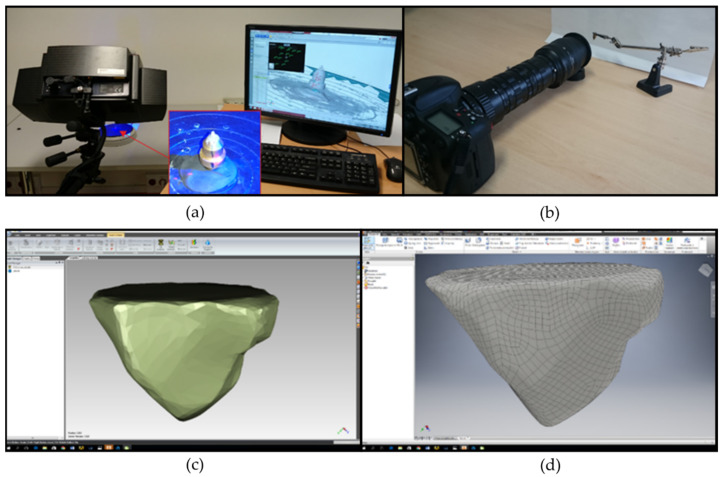
Methodology of abrasive grains shape reconstruction: (**a**) 3D scanning, (**b**) taking the macrotopography, (**c**) processing of the point clouds in the Geomagic software, (**d**) analysis of the geometric properties in Autodesk Inventor software.

The abrasive grains used for the tests were reconstructed according to the methods presented in Figure 2. The grains were scanned by means of triangulation scanner ATOS III SO (Figure 2a). In the next step, scanned point clouds were converted into the 3D grid objects using the Geomagic Design X (ver. 2019.0.1) software (Figure 2d). The correctness of shape was confirmed with previously taken macrophotography (Figure 2b). This allowed for selecting appropriate algorithms for the mesh and the point cloud conversion into the CAD (Inventor 2020) geometry (Figure 2d).

**Figure 3:**
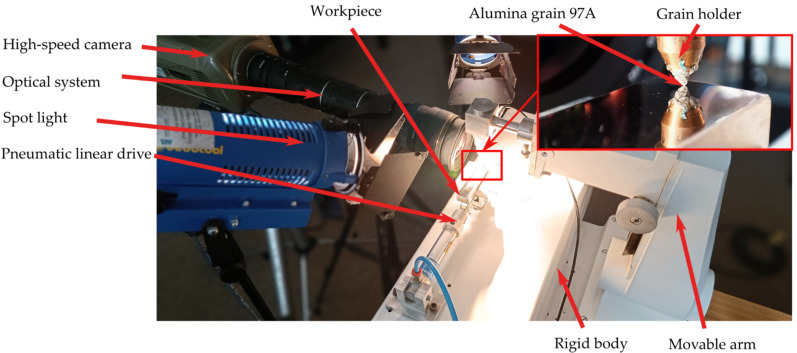
General view of the test stand for scratch tests.

In order to experimentally verify computer models of the micro-cutting process with the single abrasive grain, a test stand was built (Figure 3) and equipped with:

- Sample displacement system with pneumatic drive;
- Phantom v12.1 high-speed camera with an optical system which allowed for ×100 magnification;
- Spotlight with illuminance of 4.6 million lux.

The stand consisted of a base attached to a rigid body. The abrasive grain was placed in the holder, which was assembled into the arm, integrated with the column. The arm was equipped with a micrometric screw for an angle and the height adjustment. The pneumatic linear drive was supplied directly from a 100 L air tank with a pressure of 9 bar. The pressure was constant and allowed to obtain a cutting speed vs. = 10 m/s.

The removal of the Ti-6Al-4V titanium alloy was recorded using a Phantom v12.1 high-speed camera. The image was recorded with the resolution of 1024 × 512 pixels. The recording speed was 11,854 fps and an exposure time 41 μs. Spot lighting with an illuminance of 4.6 million lux and an optical set enabling for magnification up to ×100 was used.

In order to validate the numerical model, the cross section of the scratch was measured using a Taylor Hobson Taysurf CLI 2000 profilometer (Leicester, UK). The CLA-300 non-contact laser sensor was used for the measurement of the topography in the height range of 646 pm to 8.7 mm. Microtopographic measurements were made in 10,000 passes with 0.5 μm step. During one pass, approximately 1000 points were registered per 1 μm of length, with the table speed v = 500 μm/s. The data were processed in the TalyMap Platinum (ver. 7.4) software.

### 2.2. FEM Model

Simulations were performed in order to assess the influence of the geometric features of the abrasive grains cutting edges on the material separation process, in the form of chips or side flows. Modeling of the material separation process using the Finite Element Method allowed for studying of the phenomena occurring near the cutting edges of the abrasive grain. The simulations were carried out using the DEFORM 3D (ver. 12.1) software.

#### 2.2.1. Boundary Conditions of the Process

In the processed material, translational and rotational degrees of freedom were fixed for the nodes in the base of the material. Boundary conditions were set for the velocity of grain, which was equal to 10 m/s. The following simplifications were made: the physical properties of the grains were omitted, they were modeled as rigid bodies, and the processed material was modeled as homogeneous in its entire volume.

#### 2.2.2. Constitutive Model

Simulation studies of single grain micro-cutting processes required the application of a constitutive material model. Due to the nature of the process, the material model was selected taking into account the flow of stresses, strains, strain rates and temperature distribution. These features were very well included in the Johnson–Cook equation, which is commonly used to model materials deformations occurring in the wide range of velocity and temperature. The general form of the Johnson–Cook Equation [25,26,27,28] is as follows:

(1) $$\sigma =\left(A+B{\left(\varepsilon_{p}\right)}^{n}\right)\left(1+C\times ln\frac{{\dot{\varepsilon }}_{p}}{{\dot{\varepsilon }}_{0}}\right)\left(1-{\left(\frac{T-T_{amb}}{T_{melt}-T_{amb}}\right)}^{m}\right),$$

where *A*—initial, static yield strength; *B*—parameter of plastic strength; *ε_p_*—effective plastic strain; *n*—exponent of plastic deformation strength; *C*—material parameter specifying the impact of the intensity of plastic speed deformation; $\dot{\varepsilon_{p}}$; $\dot{\varepsilon_{0}}$ —effective plastic and reference strain rates; *T*, *Tamb*, *Tmelt*—current, ambient and melting temperatures and *m*—exponent of thermal plasticity.

The model constants are presented in Table 1 and were chosen according to Lee and Lin [26]. We have performed a series of experiments to investigate the behavior of the Ti-6Al-4V alloy during deformation at high temperature under dynamic compressive load conditions. It was presented that the flow properties of this material are temperature sensitive. For a given temperature range, the temperature sensitivity increased gradually with actual strain. The results of their experiments led to the development of constitutive equation constants that describe strains and temperature effects. They were applied to describe the plastic flow properties at high temperatures. The material constants developed by Lee and Lin have been used in many studies on orthogonal turning [27,28], in which the authors obtained high compliance with the experiments.

In order to obtain correct results of simulations of material separation processes, it is important to select the damage model. The Cockroft and Latham model [29,30] was used for numerical analyses, in which the damage coefficient increased with the deformation of the material. Material separation occurred when the damage factor reached a critical value. The damage factor was set to *D_f_* = 820 and was defined by:

(2) $$D_{f}=\int \left(\frac{\sigma^{*}}{\sigma }\right)d\varepsilon ,$$

where:

σ*—is the tensile maximum principal stress
*σ*—is the effective stress
*dε*—is the effective strain increment

The Cockroft and Latham criterion states that when the integral of the largest component of the tensile stress in relation to the plastic strain reaches the *D_f_* value, cracking and separation of the workpiece occurs [29].

#### 2.2.3. Workpiece Mesh Generation

The workpiece was discretized with 140,000 tetrahedral finite elements. In order to optimize the calculation time and the quality of the results, the auto remesh function was used, which increases the number of grid elements in the contact zone between the grain and the workpiece.

#### 2.2.4. Generate Contact or Friction

In the machining simulation processes, it is recommended to select a constant shear friction based on the constant shear hypothesis. The friction force defined for the shear constant is determined by the equation:

(3) $$f_{s}=mk,$$

where:

*f_s_*—frictional stress
*k*—shear yield stress
*m*—friction factor

In the works [31,32,33] authors demonstrated that to be able to choose a constant shear friction value, one should take into account such variables as rake angle and thermal effects. On this basis, it was found that the best results of numerical analyses could be obtained for *f_s_* = 0.8.

#### 2.2.5. Step Control

The accuracy of the simulation depends on the correct choice of the time step. Too large time step may result in solution inaccuracy, rapid mesh distortion, or a problem with convergence. On the other hand, a time step that is too small could increase the simulation time. It is recommended that the maximum displacement of any node in one step should not exceed 1/3 of the length of its element edge. The time step was determined according to the following method [34]:

1. One of the smallest elements in the workpiece was measured;
2. The maximum cutting speed of the grain was estimated;
3. The value from p. 1 was divided by the result from p. 2 and about 1/3 of this value was taken as the time step.

### 2.3. Methodology for Evaluating the Geometrical Characteristics of Abrasive Grains

**Figure 4:**
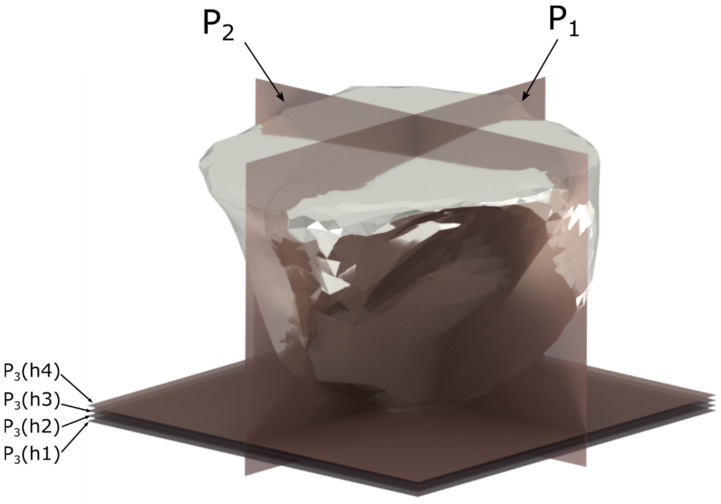
Geometrical features of the abrasive grain.

Figure 4 shows the geometrical properties of the abrasive grain. The shape of the abrasive grains was analyzed in three perpendicular planes P_1_, P_2_, P_3_, essential for the material removal process (Figure 4).

The rake angle γ of the abrasive grain was determined on the plane P_1_, parallel to the cutting direction. The apex angles *ε*_1_ and *ε*_2_ were indicated on the plane P_2_, perpendicular to the cutting direction. The opening angle *α*, the width of the contact zone *b_z_* as well as the length of the side material displacements *b_b_* = *b*_1_ + *b*_2_ were determined on the plane P_3_, parallel to the surface of the workpiece. The geometrical parameters of the grains were calculated for the variable depth of cut *h*_1_ = 25 µm, *h*_2_ = 50 µm, *h*_3_ = 75 µm, *h*_4_ = 100 µm.

#### Analysis of the Geometrical Features of the Abrasive Grain

**Figure 5:**
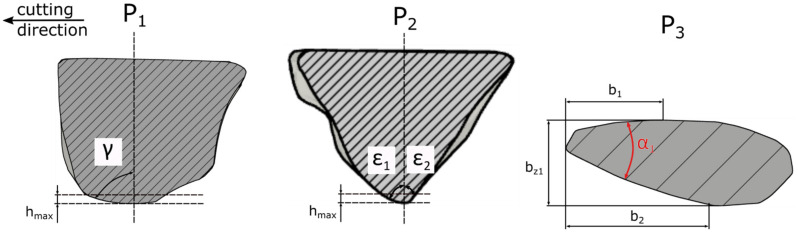
Geometrical properties of abrasive grain ZS1.

The geometry of the ZS1 abrasive grain (Figure 5) was characterized by an inclined rake surface (*α* = 45°) which angle changed with depth of cut from *γ* = −81° to *γ* = −54°. The ZS1 abrasive grain had a narrow cutting edge *b_z_*. The sum of the side cutting edges lengths (*b*_1_ and *b*_2_) was twice as long as the edges *b_z_*. The cutting angles *ε*_1_, *ε*_2_ were asymmetric with respect to the grain axis and depending on the depth of cut; they varied in the range: for *ε*_1_ from 78° to 54° and for *ε*_2_ from 74° to 40°. In the case of the described abrasive grain shape, it was expected that the machined material will move to the sides of the grain, creating side pile-ups.

**Figure 6:**
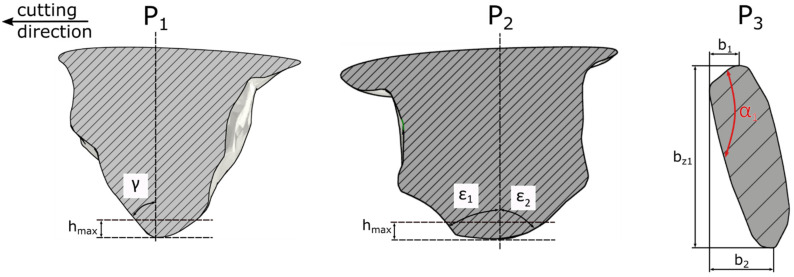
Geometrical properties of abrasive grain ZS2.

The geometry of the ZS2 abrasive grain (Figure 6) was characterized by a wide cutting edge (*α* = 134°). Its rake angle has been changing with the depth of cut from *γ* = −68° to *γ* = −42°. The sum of the lengths of two flank cutting edges *b*_1_ and *b*_2_ was more than twice of the length of the edge *b_z_*. The cutting angles *ε*_1_, *ε*_2_ were symmetrical for depth contact *h*_1_ to *h*_2_. For depths *h*_3_ and *h*_4_, the cutting angles *ε*_1_, *ε*_2_ were asymmetrical. For the depth from 0 to *h*_4_ the cutting angles varied in the range: from 84° to 37° for *ε*_1_ and from 84° to 61° for *ε*_2_. A wide cutting edge was an advantageous property of the abrasive grain [35]. That indicated its ability to form a chip and reducing the workpiece material flow to the sides of the abrasive grain.

**Figure 7:**
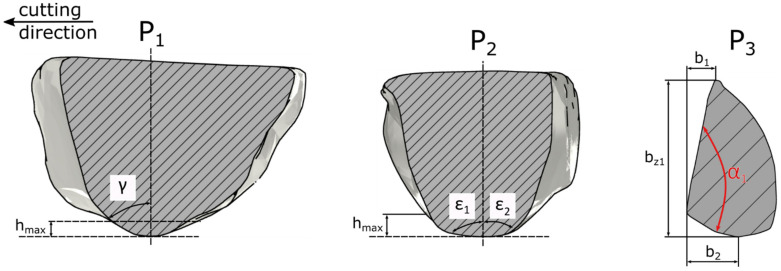
Geometrical properties of the abrasive grain ZS3.

The geometry of the ZS3 abrasive grain (Figure 7) was characterized by a wide cutting edge (*α* = 111°) and its the rake angle was varying in range *γ* = −82° to *γ* = −60° with the increase in the cutting depth. The sum of the lengths of the two flank cutting edges *b*_1_ and *b*_2_ was smaller than the width of the cutting zone *b_z_*. The cutting angles *ε*_1_, *ε*_2_ were symmetrical. For the analyzed depth of contact, the apex angles were changing in the range: from 83° to 47° for *ε*_1_ and from 81° to 48° for *ε*_2_. ZS3 abrasive grain had distinctly different lengths of the side cutting edges *b*_1_ and *b*_2_. The longer one (*b*_2_) reduced the resistance of the plastic material side flow.

The parameters determining the geometry of the abrasive grains for the variable depth of contact with the workpiece are presented in Table 2.

## 3. Results and Discussion

### 3.1. Experimental Validation of Computer Models

**Figure 8:**
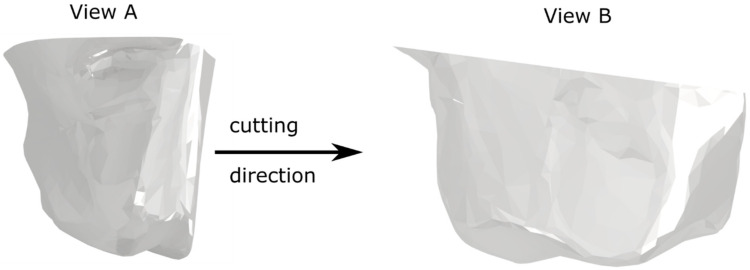
The cutting grain used for experimental validation of the numerical simulations.

The experimental validation of computer models was aimed at checking the correctness of the selected material constants of the Johnson–Cook constitutive model and the Cockroft and Latham damage model. For the tests, a grain made of alumina 97A with size F20 was used (Figure 8). The grain had a wide cutting edge with two visible apexes as shown in Figure 8 View B.

**Figure 9:**
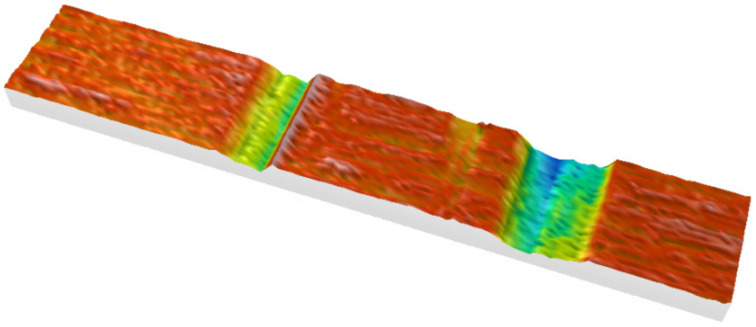

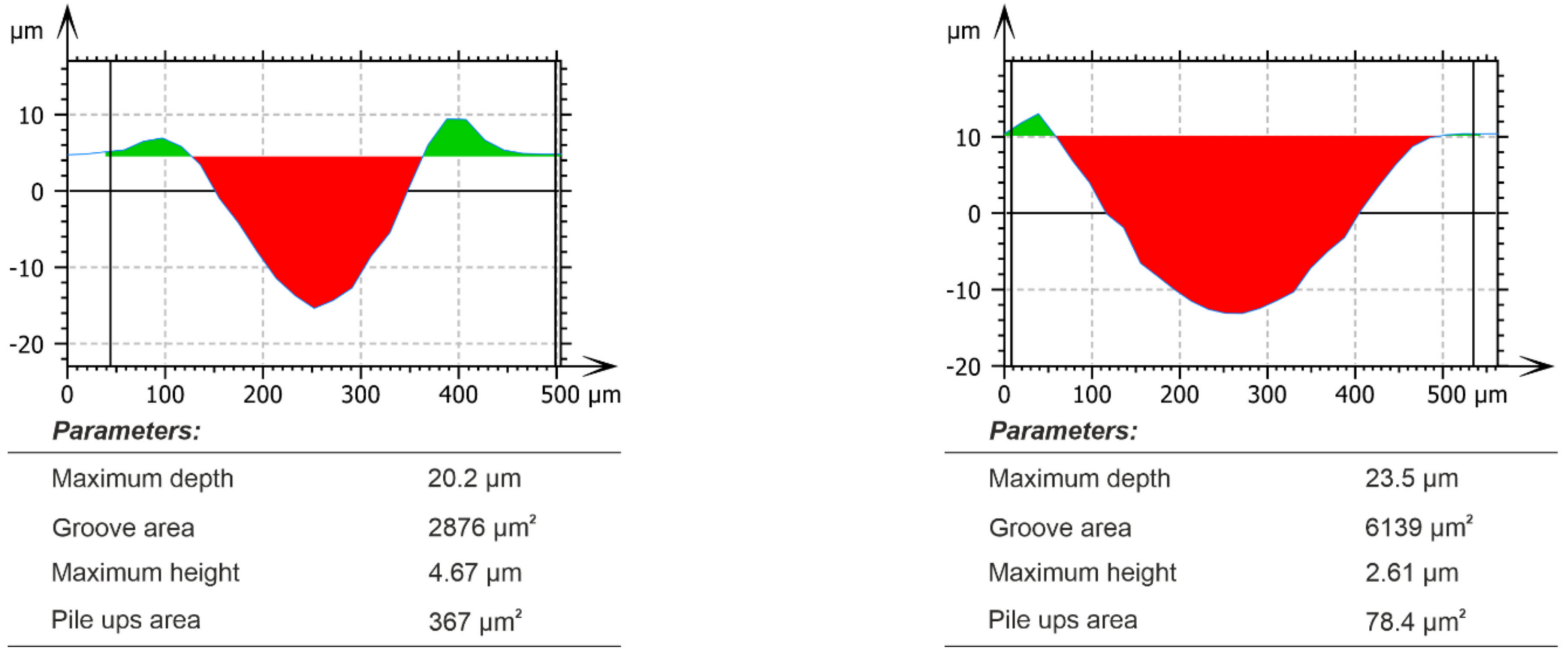
Cross-sections of the scratches—experimental results.

**Figure 10:**
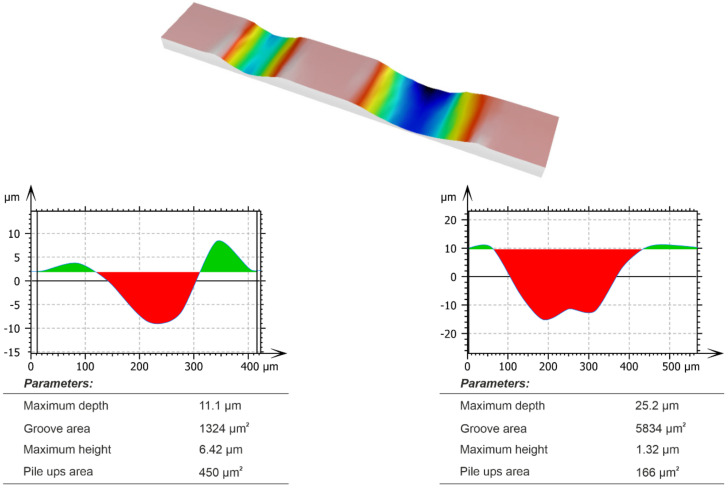
Cross-sections of the scratches—numerical results.

Figure 9 shows the result of experiment and Figure 10 shows the result of the simulation. By means of scratch test, it was proven that the correct model constants were selected. The validation was performed by comparison of the chip formation, groove and pile-ups geometric parameters.

The grain had two cutting apexes which created two scratches during the initial stage of the process. The highest convergence of results was obtained for the deeper scratch. In the case of experiments, the area of the scratch cross-section surface was equal to 6139 µm^2^. In the case of simulations, the area was equal to 5834 µm^2^. This resulted in a compliance level of 95%. A much worse result, about 50% of compliance, was obtained for the scratch cross section area, calculated after the test with smaller depth of cut. It was found that the lower consistency of results, for scratch with a smaller depth, was the result of inaccuracy of the grain spatial orientation in experimental and the simulations. The angle deviation of the abrasive grain, relative to the workpiece, affected the interaction between cutting edges and the workpiece.

**Figure 11:**
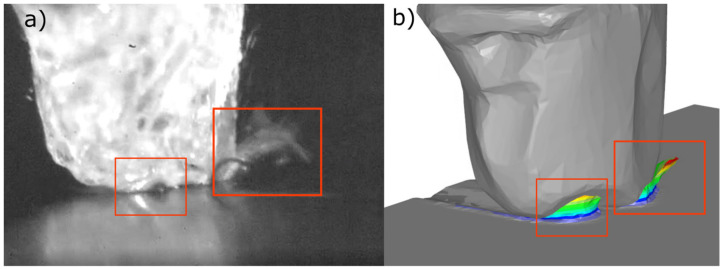
Frame from the high-speed camera (**a**) and the simulation process (**b**) as a part of the validation method.

After the comparison of the cross-sectional areas of the pile ups (Figure 9 and Figure 10), it was noticed that their compliance for the scratch with a smaller depth of contact was 78%, and for the scratch with a greater depth it was about 50%. The reason of these differences was the fracturing of the abrasive grain vertexes, visible in the pictures recorded with a high-speed camera (Figure 11a).

Experiments results (Figure 11a), of the micro-cutting process with a single abrasive grain, confirmed the correct selection of the material models and the computer simulation boundary conditions (Figure 11b). The picture recorded using the high-speed camera (Figure 11a) shows the formation of a chip (marked in red frames), which shape and direction were similar to those in the simulations. The validation shows that the FEM model presented in Section 2.2 was correct and the results of the simulations presented below were representative.

### 3.2. The Analysis of Material Removal Process by the Grains with Different Shapes

**Figure 12:**
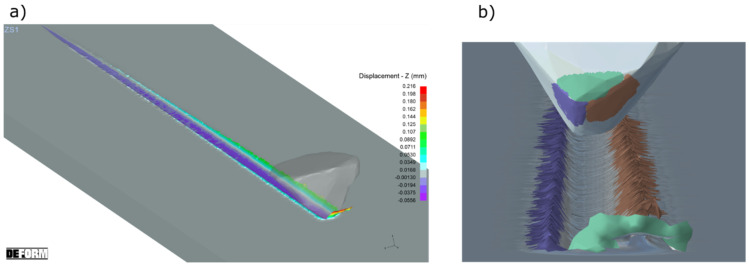
The results of the computer simulation of the cutting process with ZS1 grain (**a**) and the colored areas which caused the plastic flows of the processed material (**b**).

**Figure 13:**
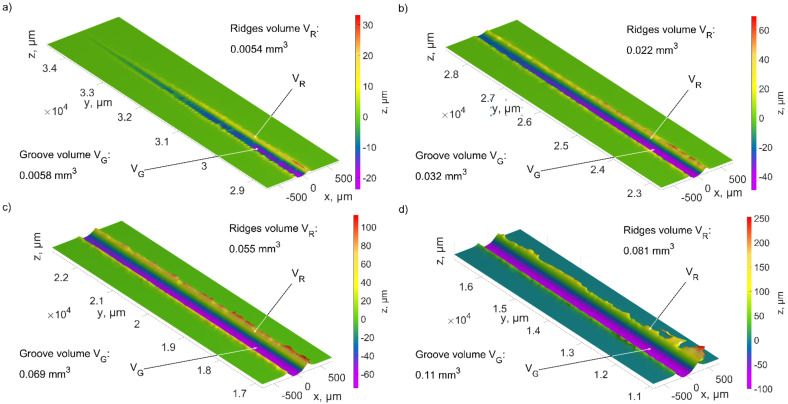
The values of the scratch volume and pile ups after the ZS1 grain cutting process for the depth of cut: (**a**) from 0 to 25 µm, (**b**) from 25 µm to 50 µm, (**c**) from 50 µm to 75 µm, (**d**) 75 µm to 100 µm.

**Figure 14:**
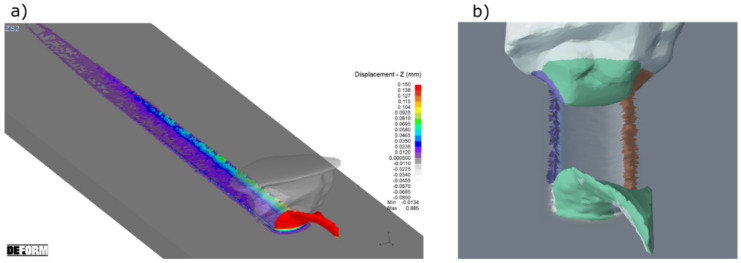
The results of a computer simulation of the cutting process with ZS2 grain (**a**) and the colored areas which caused the plastic flows of the processed material (**b**).

**Figure 15:**
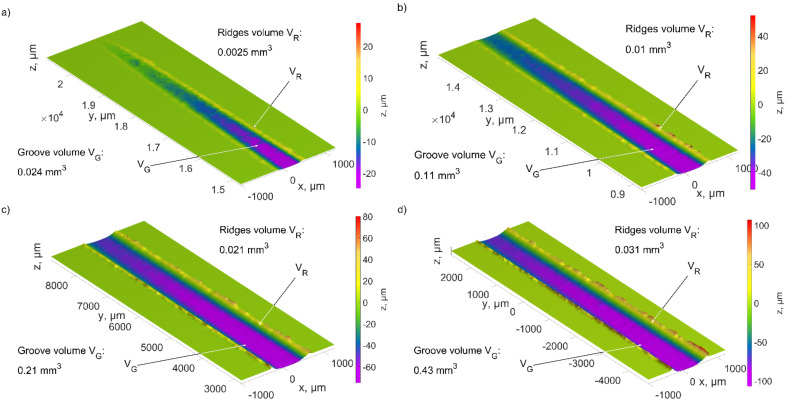
The results of the scratch volume and pile-ups after the ZS2 grain cutting process for the depth of cut: (**a**) from 0 to 25 µm, (**b**) from 25 µm to 50 µm, (**c**) from 50 µm to 75 µm, (**d**) 75 µm to 100 µm.

**Figure 16:**
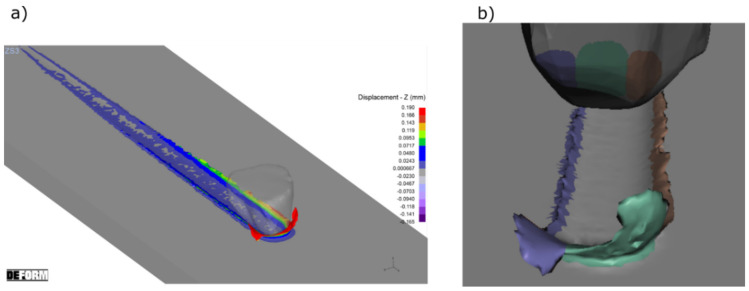
The results of a computer simulation of the cutting process with ZS3 grain (**a**) and the colored areas which caused the plastic flows of the processed material (**b**).

**Figure 17:**
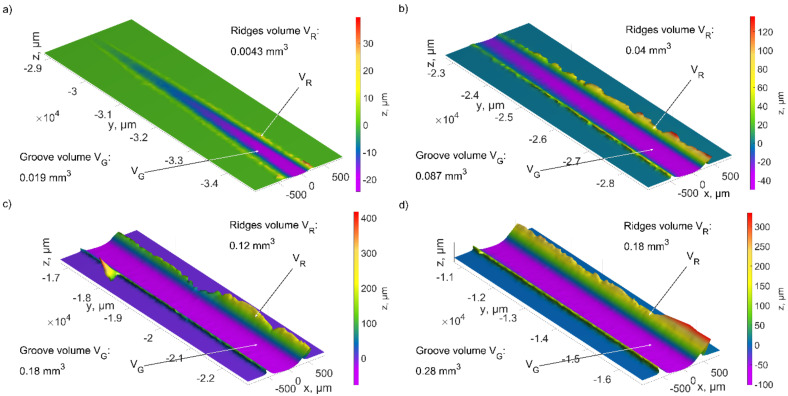
The results of the scratch volume and pile ups after the ZS3 grain cutting process for the depth of cut: (**a**) from 0 to 25 µm, (**b**) from 25 µm to 50 µm, (**c**) from 50 µm to 75 µm, (**d**) 75 µm to 100 µm.

The results analysis of micro-cutting with a single abrasive grain shows the differences in the process of the pile ups and the chip formation (Figure 12, Figure 13, Figure 14, Figure 15, Figure 16 and Figure 17). The geometrical features of the abrasive grains used in the simulations (Figure 5, Figure 6 and Figure 7), described in Section 3.1, affected the plastic side material flow.

Figure 12b shows different areas of the grain which affected the material flow. The inclined geometry of the ZS1 abrasive grain, with an opening angle *α* = 45°, a narrow cutting edge *b_z_*, long side edges *b*_1_ and *b*_2_, moved the material sideways. The area marked in brown corresponds to the longer side edge *b*_2_. It caused the displacement of larger material volumes, comparing to the shorter one *b*_1_ (purple area). Large rake angles occurred for the grain depth from 0 to 75 µm, and caused the displacement of the processed material to the sides. Their value was changing in the range *γ* = −81° to *γ* = −70°. Only a small volume of the processed material was removed in the form of chips (green area). That was confirmed by the pile ups and groove volumes, as shown in Figure 13.

The size of the side material pile ups increased as the grain moved into the material (Figure 13). In the initial contact stage, between the abrasive grain and the workpiece, plastic deformation was observed the most often (Figure 13a). The ratio of the pile-up volume to the groove volume was 0.93. The inclined grain shape was the reason why the machined material moved mainly to the sides. The depth of the contact increased, and the ratio was (min (*V_R_*/*V_G_*) = 0.69). As a result, a small chip volume was formed, and the cutting efficiency was low.

The geometry of the ZS2 abrasive grain was different because of large value of the opening angle *α* = 111° and wide edge *b_z_*. The sum of the side edges lengths (*b*_1_ and *b*_2_*)* was two times smaller than the width of the cutting edge *b_z_*. The areas shown in Figure 14b were responsible for the lateral flow of the processed material (marked in brown and purple). The smaller value of the rake angle was equal to *γ* = −42° and occurred for the grain contact depth *h* = 100 µm. Wide edge *b_z_* caused the formation of wide chip (Figure 14). The volume of the material removed in the form of chip was 100 times higher than the volume of the pile-ups (Figure 15).

The material removal process for ZS2 grain was stable regardless of the contact depth. The shape of the cutting zone caused the plastic material flow in front of the abrasive grain and then the chip was separated. The ratio of pile-up volume *V_R_* to groove volume *V_G_* was about 0.09.

The wide cutting edge of the abrasive grain ZS3, with an opening angle *α* = 111°, had long side edges, *b*_1_ and *b*_2_, the sum of which was slightly smaller than the edge width *b_z_*. The areas responsible for side material flows (marked in brown and purple in Figure 16b) were similar to the area in which chip formation occurred (green area). Large rake angles, for grain depth from 0 to 100 µm changing from *γ* = −82° to *γ* = −60°, caused the formation of pile ups as well as chip (Figure 16). This was confirmed by the results of the groove and pile-up volumes, as shown in Figure 17.

The shape of the cutting edges of the ZS3 grain was varying according to the depth of cut *h*. With a small cutting depth (up to 25 µm) the grain had a wide cutting edge. In the initial cutting stage, the volume ratio was *V_R_*/*V_G_* = 0.23. Removal efficiency decreased as the depth of contact increased. The width of the cutting zone *b_z_* decreased, whereas the length of the side cutting edges (especially *b*_1_) increased. As a result, the machined material moved to one of the sides of the abrasive grain. The ratio *V_R_*/*V_G_* increased from 0.46 to 0.63 for depths of 50 µm to 100 µm, respectively.

### 3.3. The Analysis of the Geometrical Features of the Abrasive Grain on the Efficiency of Material Removal

The geometric parameters of the cutting edges of the abrasive grain affecting the material displacement in the machining zone were as follows:

- The ratio of the rake angle *γ* to the apex angle *ε* of the abrasive grain;
- The ratio of the length of the cutting zone *b_b_* (the path that the material moves to the sides of the abrasive grain) to the width of the cutting zone *b_z_*;
- The opening angle *α* of the abrasive grain;

The opening angle *α* of the abrasive grain and the coefficient *b_b_*/*b_z_* were linearly correlated. However, when analyzing the influence of the geometrical parameters of the abrasive grains [20] and the condition of the active surface of the grinding wheel [33], the parameters determining the width and length of the cutting zone are often used. This is due to the ease of their determination, often with the use of two extreme cross-section points.

The efficiency of material removal by abrasive grain was assessed using the following factor:

(4) $$k_{r}=1-V^{\prime }\left(h\right)=1-\frac{V_{R}\left(h\right)}{V_{G}\left(h\right)},$$

where: *V_R_*—volume of the lateral pile-ups formed on both sides of the cutting edge, *V_G_*—is the volume of material removed by the cutting edge, *h*—is depth of cut.

The value of the material removal efficiency *k_r_* is equal to 1 if the volume of all material is removed as a chip and the volume of the pile-ups on both sides of the cutting edge is equal to 0.

**Figure 18:**
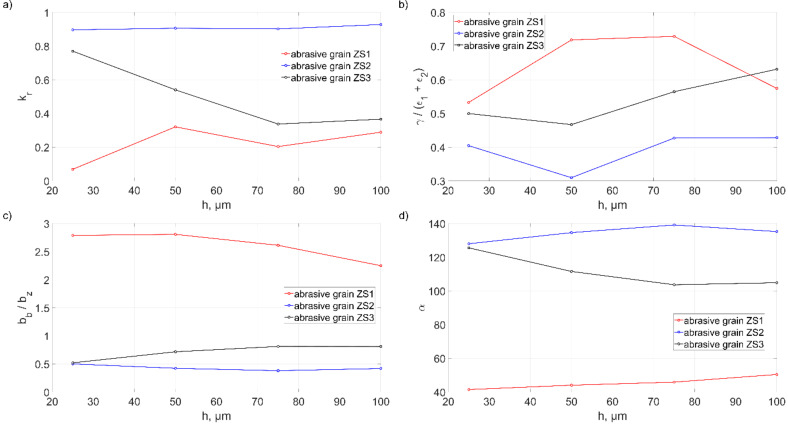
Influence of cutting depth *h* of abrasive grains with different geometry on: (**a**) the volumetric material removal coefficient *k_r_*,(**b**) the coefficient *γ*/*ε,* (**c**) the coefficient *b_b_*/*b_z_*, (**d**) the opening angle *α*.

Figure 18 shows the change in the value of the material removal efficiency coefficient *k_r_* (Figure 18a), the coefficient *γ*/*ε* (Figure 18b), the coefficient *b_b_*/*b_z_* (Figure 18c) and the opening angle *α* (Figure 18d) as a function of the depth of cut *h*.

The highest efficiency of the material removal process, irrespective of the depth of cut *h*, was observed for the ZS2 grain. That was illustrated by a small value of the coefficient *γ*/*ε* (about 0.4), a small value of the coefficient *b_b_*/*b_z_* (<0.5) and a large value of the opening angle *α* (average 134°). The smallest efficiency of the material removal process was noted for ZS1 grain. This process was dominated by lateral displacements of material which was deposited in the form of pile-ups. The nature of these interactions was illustrated by the high value of the coefficient *γ*/*ε* (0.64 on average), the high value of the coefficient *b_b_*/*b_z_* (>> 1) and the sharp opening angle *α* (45°). The ZS2 grain had variable efficiency, depending on the depth of the cut *h*, correlated with the change of parameters characterizing the geometric parameters of the cutting edge.

On the basis of the value of the coefficient *b_b_*/*b_z_*, it was concluded that for *b_b_*/*b_z_* < 1, the material was removed in the form of large, width chips and small-volume pile-ups were formed. If the ratio *b_b_*/*b_z_* = 1, the material forms the chips with small width and the pile-ups have considerable volume. If *b_b_*/*b_z_* > 1, small chips occur and large-volume pile-ups appear.

The presented coefficients of the volumetric efficiency of material removal *k_r_* and describing the features of the cutting edges, the coefficient *b_b_*/*b_z_* and *α* as a function of the depth of cut *h*, describe well the influence of the geometrical features of the grains on the efficiency of the cutting process. The shape of the ZS1 abrasive grain, the cutting edge of which has a wedge shape (*α* = 45°) and long side cutting edges *b*_1_, *b*_2_ (ratio *b_b_*/*b_z_* > 1), which causes lateral displacement of the material, is characterized by low material removal efficiency. The geometry ZS2 grain has resulted in the advantageous features of the cutting edges. The wide cutting edge *b_z_* with large opening angle (*α* = 134°) and short side cutting edges *b*_1_ and *b*_2_ limited the pile-up formation. A much larger volume of material was removed as a chip. It was also presented in the results of the volumetric material removal efficiency.

Using the experimental data, relationships were developed that determine the impact of individual parameters describing the geometry of the cutting zone on the efficiency of material removal *k_r_*. The following function was adopted for modeling:

(5) $$y=e^{a_{1}\cdot x^{a_{2}}},$$

The value of the material removal process efficiency factor *k_r_* varied from 0 to 1. The adopted mathematical model well described the physical nature of the process. For small values of the coefficient *γ*/*ε* and *b_b_*/*b_z_* and large values of the opening angle *α*, the value of the *k_r_* factor had a maximum value of 1. In these cases, the material was mainly removed in the form of chips. For large values of the coefficient *γ*/*ε* and *b_b_*/*b_z_* and small values of the opening angle *α*, the machining efficiency tends to zero. In this case, the material moves to the sides of the abrasive grain and plastic deformation dominates.

**Figure 19:**
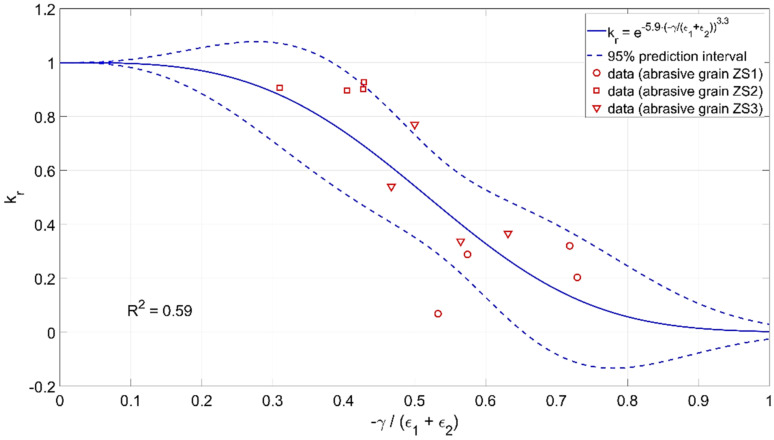
Influence of the ratio of the rake angle *γ* to the apex angle *ε* of the abrasive grain on the material removal efficiency *k_r_*.

**Figure 20:**
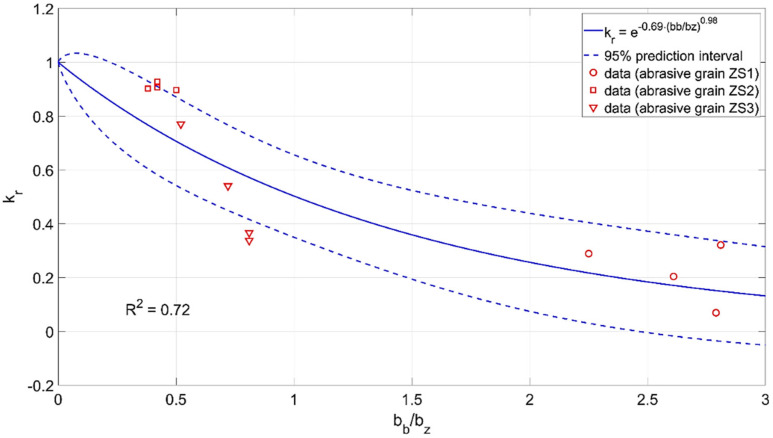
Influence of the coefficient *b_b_*/*b_z_* of the abrasive grain on the material removal efficiency *k_r_*.

**Figure 21:**
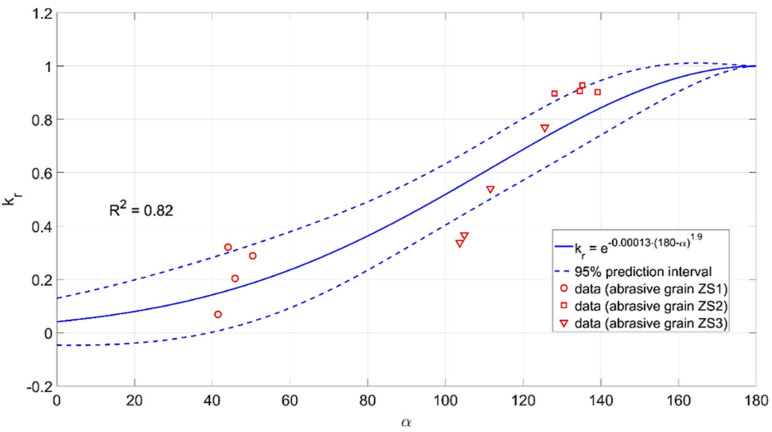
Influence of the opening angle *α* of the abrasive grain on the material removal efficiency *k_r_*.

The modeling results are shown in Figure 19, Figure 20 and Figure 21.

The parameters of the developed models are presented below:

- $k_{r}\left(\frac{\gamma }{\varepsilon }\right)=e^{-0.59\cdot {\left(\frac{-\gamma }{\varepsilon }\right)}^{3.3}}$
- $k_{r}\left(\frac{b_{b}}{b_{z}}\right)=e^{-0.69\cdot {\left(\frac{b_{b}}{b_{z}}\right)}^{0.98}}$
- $k_{r}\left(\alpha \right)=e^{-0.00013\cdot {\left(180-\alpha \right)}^{1.9}}$

The above dependencies made it possible to estimate the efficiency of material removal by an abrasive grain cutting blade. This allowed both the assessment of the impact of various geometrical features of the abrasive grains on the material displacement in the machining zone as well as the selection of the geometrical parameters of the grains that intensify the material removal processes.

The developed dependencies may constitute the basis for the design of abrasive tools and the influence of the grinding wheel dressing parameters on the material removal efficiency. They can also form the basis of systems for monitoring the condition of the active surface of grinding wheels and systems for making decisions about its durability.

## 4. Conclusions

The complexity of the grinding process causes a search for new methods of tool condition assessment and machining monitoring, which will allow to determine their effectiveness when applied to selected machining methods. The shape of the cutting blades of the abrasive grains has an influence on the material separation process in the machining zone. As a result of the experiments and numerical analyses of the grinding process with abrasive grains of the Ti-6Al-4V titanium alloy, it can be concluded that:

- Modeling of the cutting process with a single abrasive grain using FEM enabled the analysis of lateral ridges and chip formation process in the cutting with a single abrasive grain.
- Based on the value of the *b_b_*/*b_z_* ratio, it can be concluded that if: *b_b_*/*b_z_* < 1, the material will be removed in the form of a chip with a large width, small-volume ridges will be produced, the cutting efficiency will be high; if *b_b_*/*b_z_* = 1, the material will be removed in the form of a chip with a small width, there will be lateral ridges of considerable volume; if *b_b_*/*b_z_* > 1, a small volume of chip should be expected, large-volume ridges will be produced, and the cutting efficiency will be low.
- The opening angle α of the abrasive grain allowed to assess the effectiveness of material removal. A small opening angle *α* of up to 60° resulted in increased lateral material flows and thus low cutting efficiency. With large opening angles of 120–180°, the material was deposited as a wide chip, significantly increasing the efficiency of the material removal process.
- The developed models of the relationship between the geometrical parameters of the cutting blades (rake angle *γ*, apex angle *ε*, opening angle *α*, width *b_z_* and the length of the cutting zone *b_b_*) allowed us to determine the material removal efficiency for abrasive grains with different geometries.

The developed dependencies allow for their use in modeling procedures of abrasive machining processes and prediction of the topography of machined surfaces. The assessment of the geometrical features of the cutting blades of the abrasive grains provides the basis for the assessment of the condition of the grinding wheel active surface after the dressing process. It is possible to select appropriate dressing parameters that will allow the grains to be shaped in such a way that the grains oriented in the cutting direction have wide cutting edges. The use of the developed dependencies of the influence of the geometrical parameters of the abrasive grains on the material removal efficiency may be useful in the design of abrasive tools, e.g., abrasive foils, engineered grinding wheels.

## Figures and Tables

**Table 1:** Parameters of the material used in the simulations and experiment [26].

Material	A, MPa	B, MPa	C	n	M
Ti-6Al-4V	782.7	498.4	0.028	0.28	1.0

**Table 2:** Geometric parameters of tested abrasive grains.

Grain	*Depth of Cut*	*b*_1_ [mm]	*b*_2_ [mm]	*b*_z*1*_ [mm]	$\frac{b_{1}+b_{2}}{b_{z1}}[\mathrm{mm}]$	*γ* [°]	*ε*_1_ [°]	*ε*_2_ [°]	*α* [°]
**ZS1**	h_1_	0.20	0.33	0.19	2.79	−81	78	74	41
	h_2_	0.23	0.50	0.26	2.81	−74	63	40	44
	h_3_	0.27	0.54	0.31	2.61	−70	57	39	46
	h_4_	0.30	0.51	0.36	2.25	−54	54	40	50
**ZS2**	h_1_	0.09	0.19	0.56	0.50	−68	84	84	128
	h_2_	0.11	0.19	0.71	0.42	−48	78	77	135
	h_3_	0.09	0.21	0.79	0.38	−47	37	73	139
	h_4_	0.12	0.24	0.86	0.42	−42	37	61	135
**ZS3**	h_1_	0.10	0.16	0.50	0.52	−82	83	81	126
	h_2_	0.13	0.30	0.60	0.72	−64	78	59	112
	h_3_	0.20	0.36	0.69	0.81	−61	59	49	104
	h_4_	0.19	0.41	0.74	0.81	−60	47	48	105

## Data Availability

Data sharing is not applicable to this article.
